# Experimental study on electromagnetic-assisted ZnO nanofluid flooding for enhanced oil recovery (EOR)

**DOI:** 10.1371/journal.pone.0193518

**Published:** 2018-02-28

**Authors:** Muhammad Adil, Keanchuan Lee, Hasnah Mohd Zaid, Noor Rasyada Ahmad Latiff, Mohamad Sahban Alnarabiji

**Affiliations:** 1 Departement of Fundamental and Applied Sciences, Universiti Teknologi PETRONAS, Bandar Seri Iskandar, Tronoh, Perak, Malaysia; 2 Departement of Chemical Engineering, Universiti Teknologi PETRONAS, Bandar Seri Iskandar, Tronoh, Perak, Malaysia; Institute of Materials Science, GERMANY

## Abstract

Recently, nano-EOR has emerged as a new frontier for improved and enhanced oil recovery (IOR & EOR). Despite their benefits, the nanoparticles tend to agglomerate at reservoir conditions which cause their detachment from the oil/water interface, and are consequently retained rather than transported through a porous medium. Dielectric nanoparticles including ZnO have been proposed to be a good replacement for EOR due to their high melting point and thermal properties. But more importantly, these particles can be polarized under electromagnetic (EM) irradiation, which provides an innovative smart Nano-EOR process denoted as EM-Assisted Nano-EOR. In this study, parameters involved in the oil recovery mechanism under EM waves, such as reducing mobility ratio, lowering interfacial tensions (IFT) and altering wettability were investigated. Two-phase displacement experiments were performed in sandpacks under the water-wet condition at 95°C, with permeability in the range of 265–300 mD. A crude oil from Tapis oil field was employed; while ZnO nanofluids of two different particle sizes (55.7 and 117.1 nm) were prepared using 0.1 wt. % nanoparticles that dispersed into brine (3 wt. % NaCl) along with SDBS as a dispersant. In each flooding scheme, three injection sequential scenarios have been conducted: (i) brine flooding as a secondary process, (ii) surfactant/nano/EM-assisted nano flooding, and (iii) second brine flooding to flush nanoparticles. Compare with surfactant flooding (2% original oil in place/OOIP) as tertiary recovery, nano flooding almost reaches 8.5–10.2% of OOIP. On the other hand, EM-assisted nano flooding provides an incremental oil recovery of approximately 9–10.4% of OOIP. By evaluating the contact angle and interfacial tension, it was established that the degree of IFT reduction plays a governing role in the oil displacement mechanism via nano-EOR, compare to mobility ratio. These results reveal a promising way to employ water-based ZnO nanofluid for enhanced oil recovery purposes at a relatively high reservoir temperature.

## Introduction

For several years, oil has been produced from the earth using a variety of production techniques, typically occurring in three phases: primary, secondary and tertiary oil recovery [1]. Primary oil recovery is constrained to hydrocarbons that naturally rise to the surface from the existing displacement energy in a reservoir, such as gas-cap drive, solution-gas drive, and natural water drive, etc. [2]. In secondary production, water/gas is commonly injected to re-pressurize the target formation, which provide the energy as well as mobility to sweep the residual oil to the wellbore for recovery. Tertiary processes use chemicals, miscible gases, and/or thermal energy to mobilize the additional oil after the secondary recovery process [3]. There are some mechanistic distinctions between secondary and tertiary processes. In secondary processes, the injected fluids act as a supplement to the existing natural energy in the reservoir to displace oil. The recovery efficiency primary depends on the mechanism of pressure maintenance. However, the injected fluids in tertiary recovery processes, also known as enhanced oil recovery (EOR) processes, interact with the reservoir rock/oil system. These interactions can be resulted in lower interfacial tensions (IFT), oil viscosity reduction, oil swelling, wettability modification, and/or favorable phase behavior.

**Fig 1:**
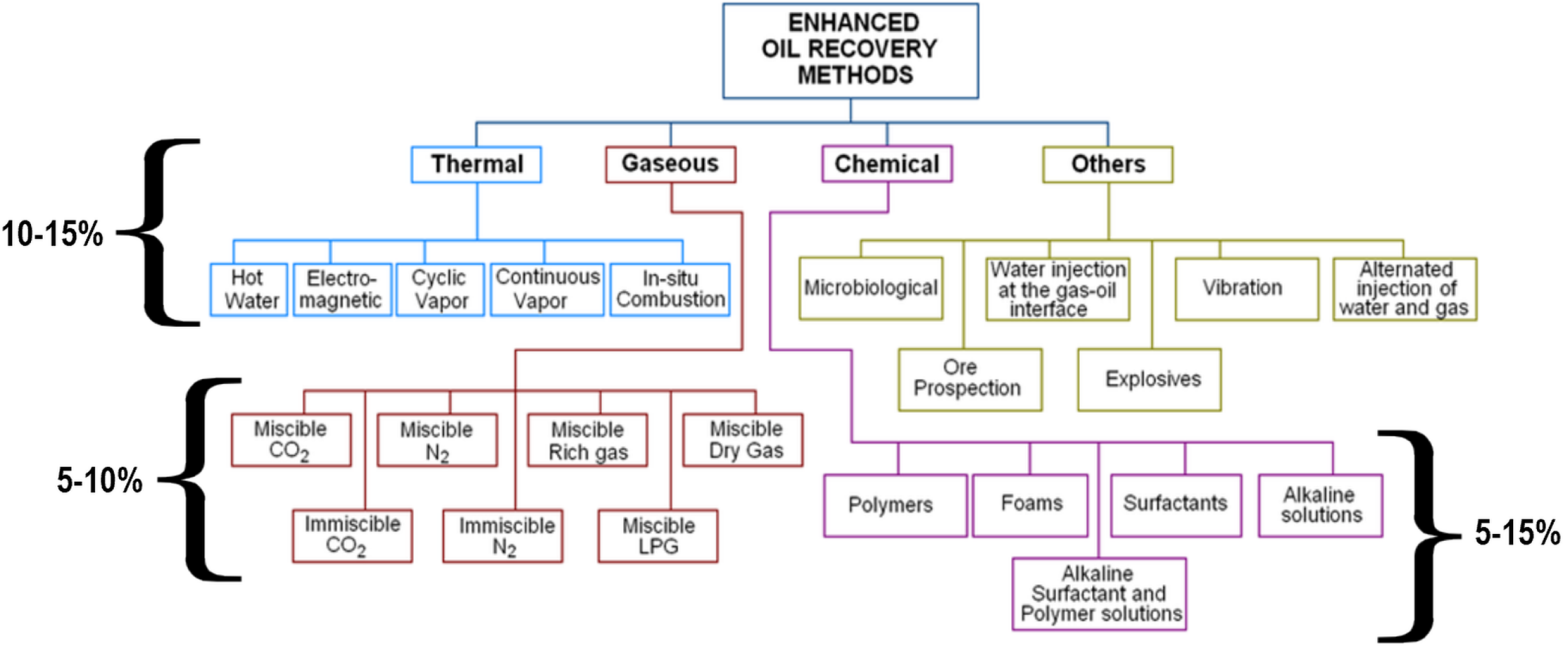
Various available EOR methods, with their typical percentage incremental recovery [8–10].

As depicted in Fig 1, various potential EOR methods can be considered in the screening stage to fit in the reservoir conditions. However, the oil reservoirs concealed in deep water possess a drastic environment that would lead to several challenges and downfall of traditional EOR techniques [4, 5]. In chemical (i.e. surfactant flooding) and gas (i.e. CO_2_) EOR methods, the change of properties of injection fluids under the extreme condition is one of the major challenges. Moreover, chemical processes are often constrained by the high cost of chemicals, possible formation damages, and losses of chemicals [6, 7]. As the depth of the reservoir increases, the thermal methods also become ineffective due to the high energy cost, as well as heat loss from generation source to undesired reservoir levels. Therefore, less expensive, more efficient, and environmentally friendly EOR methods are greatly needed. Nanoparticles offer innovative pathways to address the unsolved challenges, which show some useful characteristics as EOR agents. The significant advantage of nanoparticles is that they can be tailor-made in a flexible manner using combinatorial chemical approaches and provides therefore an approach to transcend the current EOR technology.

In the past decade, most studies have proven that the nanoparticles (NPs) offer a potential for future enhanced oil recovery (EOR) processes where silica-based NPs have been commonly used [11–13]. However, with the advancement in nanosciences, the use of metal oxide NPs have proven their ability as EOR agents and their advantages over SiO_2_ NPs. For instance, these nanoparticles have been developed to promote wettability alteration, reduction of viscosity ratio, stabilization of foam or emulsion and interfacial tension reduction–in some case involving the application of an external electric or magnetic field. Ogolo et al. [14] investigated the performance of eight NPs (oxides of zinc, aluminum, magnesium, iron, zirconium, nickel, tin, and silicon) and four base fluids (distilled water, brine, ethanol, and diesel oil) for EOR. These results showed that Al_2_O_3_ NPs suspended in brine and distilled water had the tendency to improve oil recoveries through reduction of viscosity ratio. Hendraningrat et al. [15] also studied Al_2_O_3_ and TiO_2_ NPs, and concluded that the highest cumulative oil recovery was achieved by using TiO_2_. In heavy oil, Ehtesabi et al. [16] used titanium oxide (63±8 nm) to enhance the oil (> 40 cP) recovery in flooding experiments on a laboratory scale. Hendraningrat and Torseater [17] has furthermore described a method to enhance oil recovery by wettability alteration using aluminum, silicon, and titanium oxide nanoparticles. They concluded that oil recovery increased (and the contact angle decreased towards more water-wet system) as nanoparticles size decreased.

**Fig 2:**
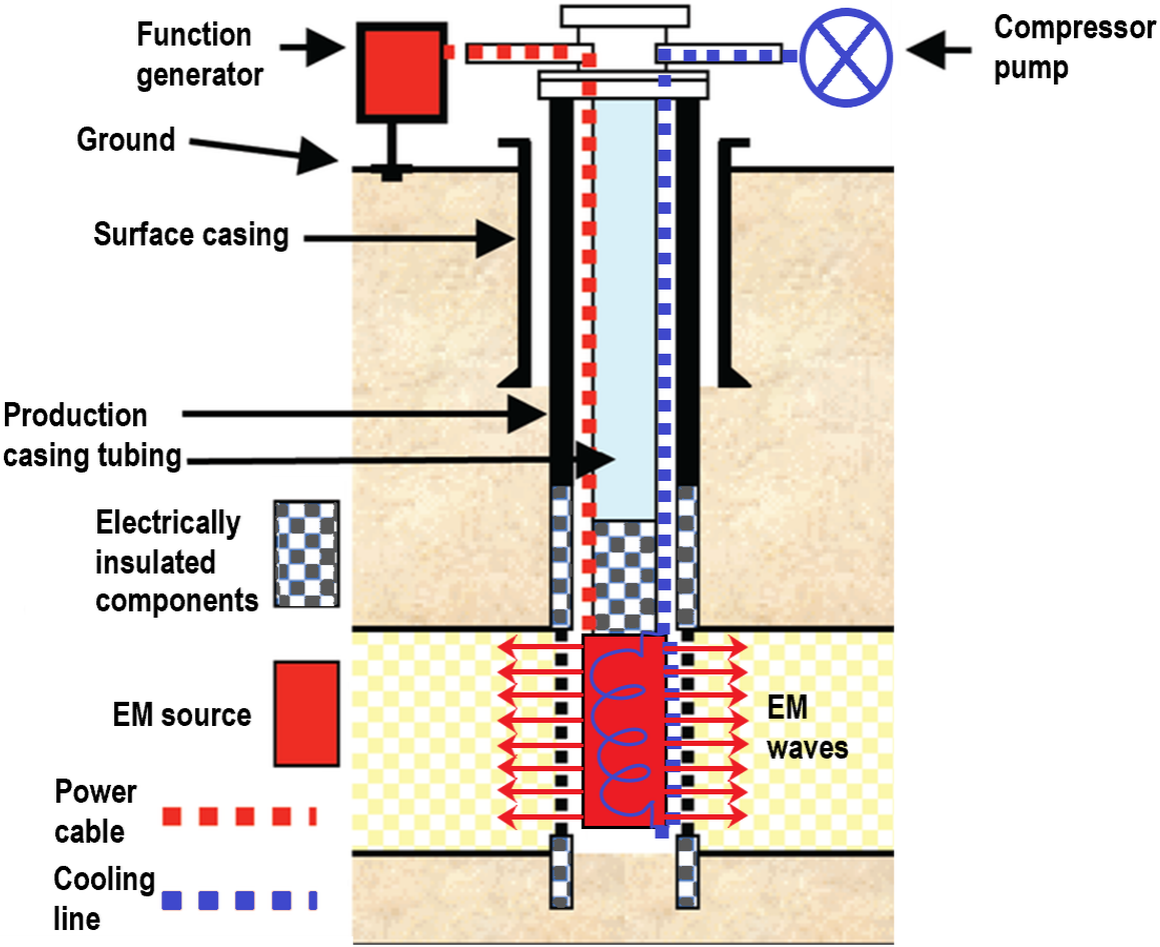
Schematic view of an EM-based EOR setup in field level.

Meanwhile, Haroun et al. [18] proposed a new EOR method, called electric EOR (EEOR). In this process, electric current (of about 2 V/cm) was applied to increase the oil recovery of nanofluid (FeO, CuO, and NiO) flooding by 9–22%. Latiff et al. [19] also have demonstrated more than 30% increase in the incremental recovery with ZnO and Al_2_O_3_ nanofluids under the application of 60 MHz EM wave. Besides, it has been shown experimentally that oil droplets surrounded by NPs can be deformed under the influence of an external electric field [20]. This phenomenon may in part be responsible for the EEOR-induced improved oil recovery. Based on these studies, it is clear that the oil displacement mechanism via EEOR is not entirely understood yet. Therefore, to maximize oil recovery due to EEOR, it is crucial to have a clear depiction of parameters that may influence the oil recovery mechanism. For this purpose, dielectrics (such as ZnO, Al_2_O_3_) are of great interest as suitable metal oxides for EEOR application due to their high dielectric loss that render the particles as the surface-active agent [21]. The surface-active agents suspended in a liquid undergo polarization when an external electric field is applied, providing the possible oil recovery mechanism including (i) the deformation of oil droplet which increases the surface area for the adsorption of additional particles, and consequently causes a reduction in interfacial tension; (ii) the increment in rate of wettability alteration which lead to an increase in surface free energy; (iii) the improvement of mobility ratio due to electrorheological effect (ER) which increases the viscosity of nanofluids. Fig 2 also presents the feasibility of EEOR in field scale, with an EM source installed inside an injection well. Signal generator is placed on the ground surface and provides power to the EM antenna installed down hole. EM waves are transmitted, through the perforated metallic casing, by the interaction with the water molecules as they are polar in nature. A suitable air/water cooled system is also installed inside the injection well which is supplied with cold water or cool air from the compressor pump installed on the ground surface. This is done to prevent the source from getting damaged because of exposure to high temperature environment for a long period.

In this study, laboratory experiments were designed to determine the relationship between governing parameters and EM-assisted oil recovery due to nanofluids. A two-phase sandpack flooding experiments were conducted by injecting the surface-modified ZnO nanofluids as a tertiary process (nano-EOR), at reservoir temperature of 95°C. An injection cycle scheme was developed to show the great potential of using ZnO nanofluids in EEOR method. The crude oil/brine/rock (COBR) interaction was determined using contact angle on quartz plates, which were used as the solid-phase, to establish the possible oil displacement mechanism under EM waves. In addition, fluid-fluid interactions (oil/brine/nanofluid) were evaluated using interfacial tension results, along with viscosity ratio between NFs and crude oil.

## Materials and methods

### Materials

ZnO nanoparticles were synthesized using sol-gel auto-combustion method [22], and were used after calcining at 500°C and 800°C having an average particle size of 55.7 and 117.1 nm respectively [23]. In this paper, these particles were denoted as ZnO@500 and ZnO@800, respectively. The analytical grade of sodium dodecylbenzenesulfonate (SDBS) from Sigma Aldrich was used as a stabilizer, without further purification. Deionized water (with σ = 18 MΩ) was used as a solvent. NaCl obtained from Fisher Scientific, was employed as salt to prepare brine of a concentration of 30000 ppm (equivalent to sea water concentration). The pH value of system was adjusted with HCl and NaOH solution by precise pH meter (Mettler Toledo, FE20-Basic). For flooding experiment, crude oil sourced from a field in Tapis (geographical coordinates of 05° 31′ 44.85 ″ N, 104° 57 ′ 20.77 ″ E) has been employed. It has density and viscosity of 0.802 g/cm^3^ and 7.5 cP, respectively at ambient condition. The 30000 ppm brine was also used as a saturation and injecting fluid. The reason is that brine is present in oil reservoirs as well as readily available in offshore fields. Table 1 shows fluid properties measurement of brine, crude oil and various nanofluid concentration at ambient condition.

**Table 1:** Fluid properties at ambient condition.

Fluid	Density (g/cm^3^)	Viscosity (cP)
Brine, NaCl 3 wt. %	1.0197	1.01
Crude oil	0.8021	7.50
SDBS 0.025 wt. %	1.0194	1.02
ZnO@500 NF	1.0204	1.05
ZnO@800 NF	1.0205	1.07
ZnO@500 NF (With EM)	1.0204	1.10
ZnO@800 NF (With EM)	1.0205	1.14

### Nanofluid preparation

The nanoparticles were dispersed in brine as the base fluid and magnetically stirred for 1 hour to produce nanoparticles suspension. Then, the appropriate amount of sodium dodecylbenzenesulfonate (SDBS) was added to the suspensions. These suspensions were agitated in an ultrasonic bath at ambient temperature for an optimum period, to attain the required concentration of nanofluids. The anionic surfactant, SDBS, was chosen as a stabilizer based on our previous stability tests [24]; where the surfactant concentrations were selected using critical micelle concentration (CMC) determination methods [25]. The pH value of the system was also adjusted by using HCl and NaOH solution to improve the quality of dispersion. These pH values were monitored by precise pH meter (FE20-Basic) from Mettler Toledo.

### Interfacial tension and contact angle measurement

**Fig 3:**
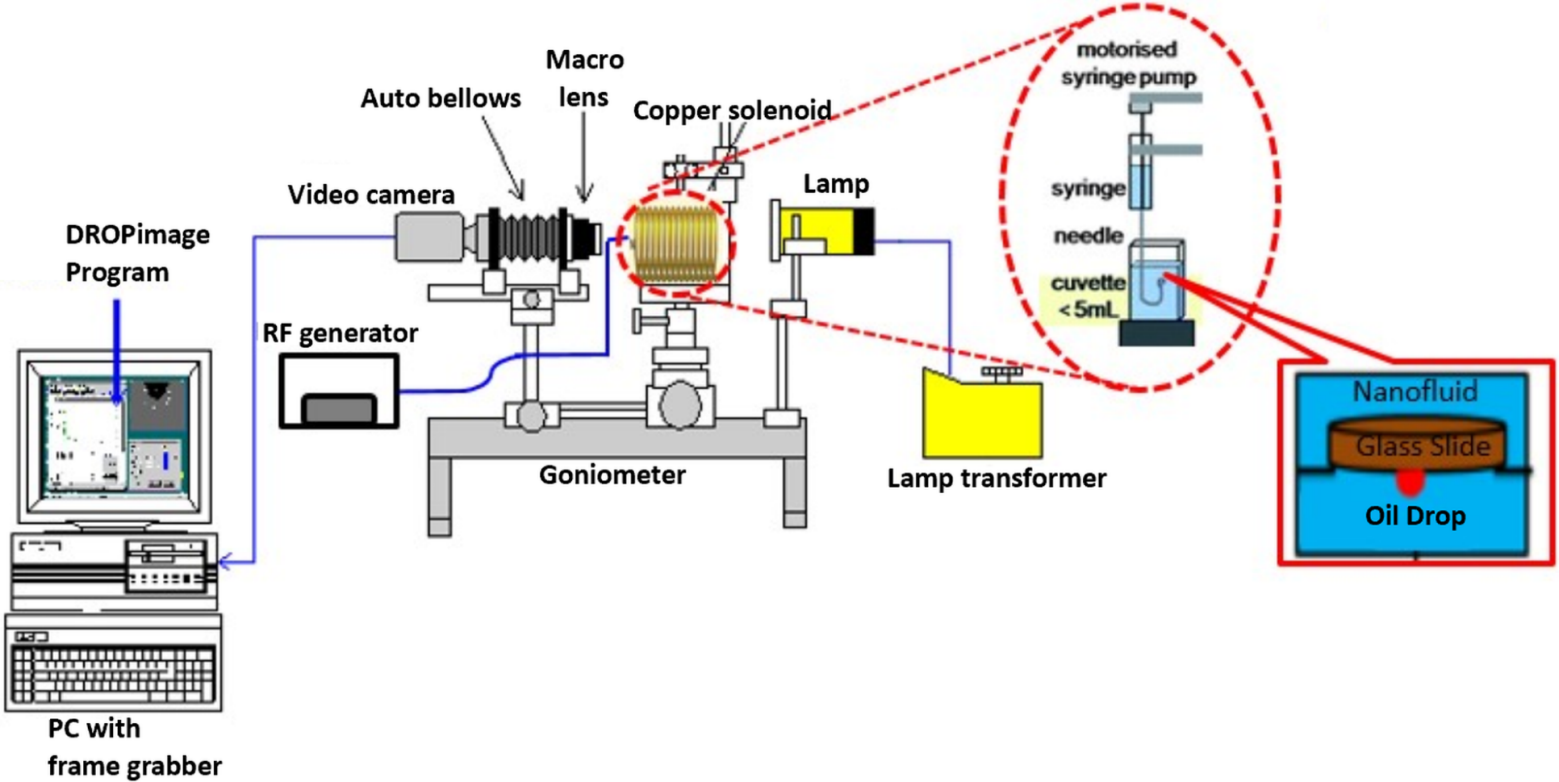
Schematic illustration of the goniometer, customized for the measurement of interfacial tension and contact angle under electromagnetic waves.

The classical method of sessile drop-shape analysis was used to determine the oil/brine and oil/nanofluid interfacial tension and the solid-nanofluid-oil three-phase contact angle. These measurements were conducted by using a Goniometer (Ramé-hart Model 260), at ambient conditions. The system consists of a glass plate as the solid, crude oil from Tapis as the oleic phase and nanofluids as the aqueous phase. The imaging setup is illustrated in Fig 3 shows a glass cell container, surrounded by a custom-built solenoid coil, located between a light source and a magnifying camera. The diameter of the coil, fixed at 7 cm due to the limit of sample dimensions, was connected with RF generator (Agilent 33500B) to generate EM waves.

As shown in Fig 3, a glass plate representing sandstone, were placed on cell filled with the nanofluid. A small drop of 24 ± 0.2 μL of the crude oil to be measured was then placed underneath the glass plate, using an inverted syringe. Since the density of oil is lower than the density of nanofluid, the oil drop had to be placed under the glass plate. The camera magnified the drop and presented an image on the connected computer. Adjustments and calibrations were made manually to get a clear and focused image of the drop and surface. Once the drop-shape profile stopped changing which indicates a constant interfacial tension, a camera was used to take pictures. This side-view profile was utilized by the software DROPimage to evaluate the interfacial tension (γ) by using the following equation

$$\gamma =\Delta \rho \;g\;R_{0}^{2}/\beta$$ (1)

where *Δρ* is the density difference between the oil drop and the surrounding medium, *g* is the gravity constant, *R*_*0*_ is the radius of curvature at the drop apex, and *β* is the shape factor.

The software DROPimage was also employed to determine the three-phase contact angle, which uses an image analysis technique. For each picture, the baseline between the solid surface and the drop must be defined by manual adjustments in the software. This enables the software to recognise the drop and to find the best matching contact angles on both sides of the drop. A mean value between the contact angles on the left and right side of each image was calculated. Then an overall mean contact angle was calculated for the entire experiment.

### Sandpack flooding setup

**Fig 4:**
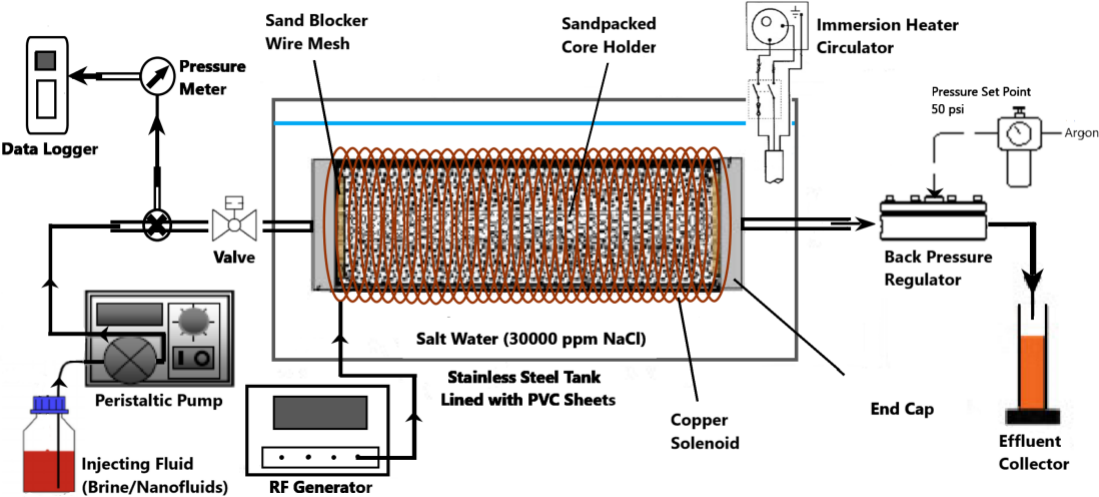
Schematic of EM-assisted experimental setup for sandpack flooding.

As depicted in Fig 4, the experimental setup assembly consists of a peristaltic pump (Masterflex L/S, Cole Parmer), a 10-bar pressure sensor (PS100, Lutron), a pressure data logger (PS9302, Lutron), a stainless-steel (SS) tank, a 100-psig back-pressure regulator (Equilibar) and measuring cylinders for effluent collection. The experimental tank was filled with 30000 ppm salt water (equivalent to seawater), inside which the sandpack core holder is placed on a plastic stand. The sanpack holder was surrounded by a custom-built solenoid coil in case of EM-assisted nanoflooding. These solenoids were specifically designed to facilitate the generation of EM waves under salt water at a laboratory scale. The inside of the experimental tank was also lined with PVC sheets to avoid the reflection of EM waves, during the experiment. A stand-alone immersion heating circulator (SC-100, Thermo Fisher Scientific) was also mounted on the tank, to provide a uniform temperature of 95°C throughout the flooding experiment. In addition, a valve was installed at the inlet; while a back-pressure regulator was installed at the outlet to provide a constant pressure of 50 psi in the sandpack. This laboratory based research was required no specific permission to perform research activities, as it doesn’t endandgered any living species.

#### Sandpack preparation

**Fig 5:**
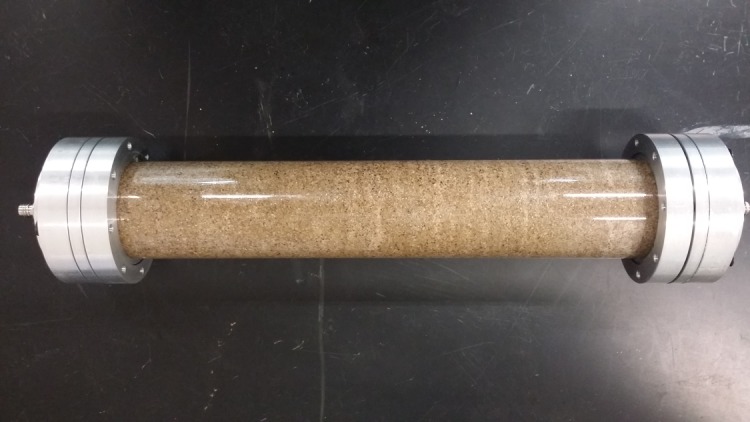
A sample of sandpacked core holder.

An acrylic core holder of 4.6 cm in diameter and 30 cm in length was used for the sandpack. The size of the core holder, is in accordance with the literature, ranging from 1–2 inches (2.5–5.1 cm) diameter and 1 ft (30 cm) long [26–28]. For each flooding test, the fresh quartz sand from Sungai Perak, Malaysia (300–425 μm mesh) was packed to ensure the identical initial status of wettability. The vertically oriented core holder, with a volume of 500 cm^3^, were uniformly packed with dry sand. The sand was compacted by filling the holder layer by layer, followed by 5 minutes of vibration. After the sand was tightly packed, a top sieve and cap were fixed. The caps on both the ends of the holder were attached with connectors for the insertion of inlet and outlet tubes. Rubber ‘O’ rings surrounded the caps were also used to seal the core holder hermetically. The holder was then vacuumed for an hour to remove any trapped air. Fig 5 shows a sample of core holder packed with 300–425 μm mesh sand.

#### Sandpack flooding procedure

The sandpack, mounted vertically, was first flooded with brine at a constant flow rate of 1 cm^3^/min to determine the porosity and permeability of sandpack (tabulated in Table 2). The porosity (*%*) of the column was calculated as the pore volume (*PV*, cm^3^) divided by the bulk volume of the column (*BV*, 500 cm^3^). Pore volume (*PV*), defined as the empty volume of the core holder, was determined by measuring the volume of brine needed to saturate the holder. Porosity (*φ*) of packed column is determined mathematically by following equations:

$$\varphi =\frac{PV}{BV}\times 100\%$$ (2)

$$PV=\frac{Saturated\;weight\;(gm)-Dry\;weight\;(gm)}{\rho_{b}}$$ (3)

$$BV=\frac{\pi }{4}d^{2}L$$ (4)

where *L* is the length of the packed column (cm^3^), *d* the diameter of the column (cm), and *ρ*_*b*_ the density of brine (g cm^-3^).

**Table 2:** Petrophysical properties of sandpacks at the initial condition.

Sanpack number#	Pore Volume (cm^3^)	Porosity (%)	Average permeability (mD)	Flooding case
1	196.13	39.33	303	Brine + SDBS
2	176.52	35.40	267	Brine + SDBS + ZnO@500
3	186.32	37.37	284	Brine + SDBS + ZnO@800
4	181.42	36.38	284	Brine + SDBS + ZnO@500 (With EM)
5	186.32	37.37	284	Brine + SDBS + ZnO@800 (With EM)

While the permeability was determined by measuring the pressure drop across the entire sandpack, and then calculated using Darcy’s law.

$$k=\frac{\mu \;L\;Q}{A\;\Delta P}$$ (5)

where *μ* is the dynamic viscosity of brine (cP), *L* the length of the packed column (cm), *Q* the flow rate of brine through the column (cm^3^ s^-1^), *A* the cross-sectional area of the column (cm^2^), and *ΔP* the difference between the inlet and outlet pressures (atm).

In the second step, the brine-saturated sandpack was flooded with the crude oil using a peristaltic pump (Masterflex L/S), and this continued until no more water was produced from the outlet. Original oil in place (*OOIP*, cm^3^) was then evaluated as the volume of oil retained in the holder, by measuring the volume of brine in the effluent. Initial oil saturation (*S*_*oi*_, %) and initial water saturation (*S*_*wi*_, %) were calculated using material balance, as follows:

$$S_{oi}=\left(\frac{OOIP}{PV}\right)\times 100$$ (6)

$$S_{wi}=\left(\frac{PV-OOIP}{PV}\right)\times 100$$ (7)

After these parameters were obtained, the sandpack flooding tests were performed horizontally inside the SS tank at reservoir temperature of 95°C, and an injection rate of 1 cm^3^ min^-1^. The injection rate is equivalent to 4 ft/day with a shear rate of 10 s^-1^, which is in accordance with the shear rates of 0.01 to 10 s^-1^ in most formations [29]. On the basis of establishment of *S*_*oi*_ and *S*_*wi*_, sandpack flooding tests began with the initial waterflooding (denoted as WF1), and the injection of brine was continued for 1 PV to achieve waterflood residual oil saturation (*S*_*or*_). This stage of WF1 also represents the secondary phase in the oil recovery process. 1 PV was set as the limitation due to high porosity & permeability of sandpack, to achieve the realistic and uniform S_or_ value among all the sandpacks. The amount of crude oil recovered after water flooding (*S*_*orwf*_, cm^3^) was measured volumetrically. Residual oil saturation (*S*_*or1*_) was calculated as follows:

$$S_{or1}=\left(\frac{OOIP-S_{orwf1}}{OOIP}\right)\times 100$$ (8)

The residual oil was then subjected to Nano-EOR as tertiary recovery process. In this step, 1 PV of nanofluids (ZnO@500 & ZnO@800) were injected in two scenarios; without EM field (denoted as NF), and with EM field (denoted as EMNF). In case of EM-assisted EOR, the solenoid as an EM-source was submerged in salt water; while connected to a RF generator (Agilent E4421B). The effluent from NF flooding was collected in fractions of 0.1 PV using a volumetric graduated cylinder. All the collected fractions correspond to the oil recovered after Nano-EOR flooding (*S*_*ornf*_, cm^3^). During the flooding, the injection pressure was also monitored at 1-minute intervals using a pressure sensor connected to a data logger. As a comparison, brine with SDBS alone was also performed as tertiary flooding. The residual oil saturation (*S*_*or2*_) after nano flooding was calculated as follows:

$$S_{or2}=\left(\frac{OOIP-S_{orwf1}-S_{ornf}}{OOIP}\right)\times 100$$ (9)

In the end, brine was re-injected (denoted as WF2) at the same condition with previous nano flooding. The effluent water was collected (*S*_*orwf2*_, cm^3^); and recorded at each 0.1 PV. The final residual oil saturation (*S*_*or3*_, %) after post flushing was calculated as follows:

$$S_{or3}=\left(\frac{OOIP-S_{orwf1}-S_{ornf}-S_{orwf2}}{OOIP}\right)\times 100$$ (10)

## Results and discussion

In the present research, three sets of sandpack flooding scheme have been conducted. In the first scheme, surfactant flooding (SDBS in brine) was carried out after water flooding as a reference study. While for the second set of flooding, the enhanced oil recovery over water flooding has been investigated using different concentrations of ZnO NPs. In the last set, ZnO NFs have been injected in conjunction with EM system, after water flooding. In these tests, the effects of ZnO NPs concentration, and their hydrodynamic sizes on the oil recovery were studied, with and without the presence of EM waves.

### Sandpack flooding tests

Five sandpack flooding tests were performed to explore the effectiveness of the nano flooding system for enhanced oil recovery by employing the method described above. The summaries of these displacement tests are presented in Table 3. Based on these results, initial water saturation (*S*_*wi*_) was greater than 25% for all the floods which indicates a water-wet characteristic of the sandpack holder; consistent with Craig’s rule of thumb [30]. According to Craig, the water fills the small pores and forms a thin water film over the rock surfaces in a water-wet system. Therefore, the *S*_*wi*_ is relatively high in a water-wet system. The variation of *S*_*wi*_ values (as presented in Table 3) at similar wettability may be caused by the change in sand distribution during sand packing which alters the absolute permeability of sandpack. However, Viksund et al. [31] observed only minor variation in ultimate oil recovery by changing of initial water saturation from 0 to 30%. Hence, it is reasonable to expect the final recovery to reach the same percentage of original oil in place. As a secondary recovery method, waterflooding (WF) is frequently used in which 1 PV of brine (3 wt. % NaCl) is injected into the sandpack to displace the oil; while the temperature and pressure of the system are maintained at 95°C and 50 psi, respectively. The oil recovery performance, as well as the pressure drop profiles, of sandpack flooding can be seen in the figures below. The average oil recoveries after WF are in the range of 54.7–57% of original oil in place (OOIP), and the residual oil saturation (*S*_*or1*_) ranges from 42.9 to 45.2% of PV. Tang and Morrow [32] studied that the oil recovery due to waterflooding (WF) increased as the temperature increased to 75°C, due to the COBR interactions which control the increase in oil recovery with temperature (i.e., the reduction in IFT). In addition, Morrow [33] also suggested that the oil recovery due to waterflooding is limited between 50–60% OOIP, which is possibly linked to disconnection and the trapping mechanism of the oil phase. As shown in these figures, water breakthrough is observed between 0.5 to 0.6 PV for all the flooding tests corresponding to a secondary recovery of 54–57% OOIP. Water breakthrough is defined as the first water produced at the outlet of the flooding system. However, only a small amount of additional oil was recovered after water breakthrough.

**Table 3:** Summary of ZnO nanofluid flooding at 95°C, without and with the electromagnetic field.

Sandpack number#	Initial Water Saturation S_wi_ (% PV)	Oil Recovery (% OOIP)			Residual Oil Saturation (% PV)			Displacement Efficiency (E_D_)	Total Oil Recovery (% OOIP)	Flooding Case
		WF1	NF	WF2	WF1	NF	WF2			
1	27.60	57.04	2.18	0.17	42.95	40.77	40.59	5.08	59.40	SDBS alone [Fig 6]
2	30.88	54.71	10.27	3.55	45.28	34.87	31.27	22.62	68.52	SDBS + ZnO@500 [Fig 8A]
3	29.15	56.06	8.59	6.66	43.93	35.34	28.67	19.56	71.32	SDBS + ZnO@800 [Fig 8B]
4	29.44	55.27	10.39	3.59	44.72	34.33	30.74	23.23	69.25	SDBS + ZnO@500 (With EM) [Fig 10A]
5	27.01	56.25	9.00	7.05	43.75	34.74	27.68	20.58	72.31	SDBS + ZnO@800 (With EM) [Fig 10B]

### Scheme I: Surfactant flooding

**Fig 6:**
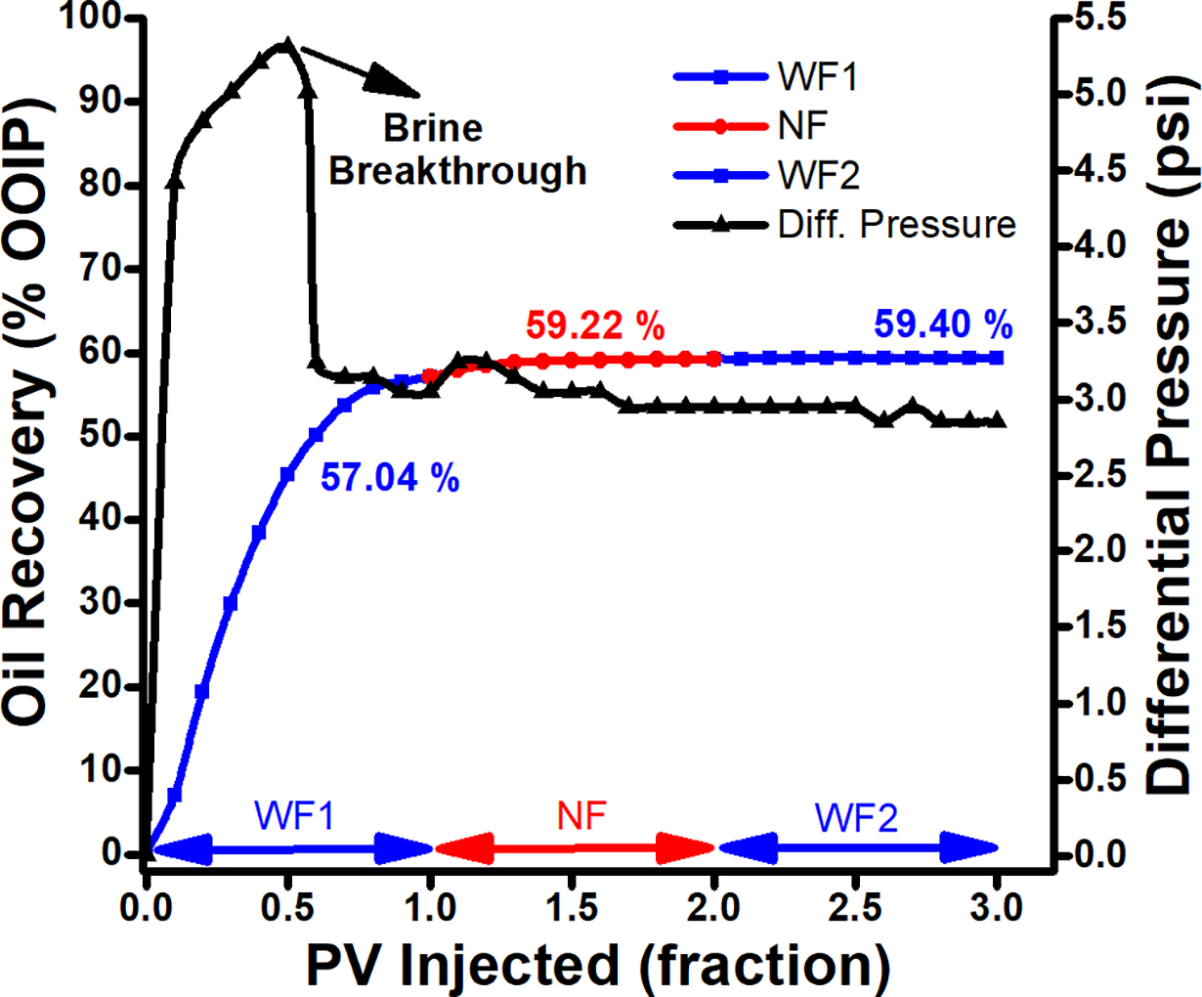
Oil recovery performance and differential pressure of SDBS surfactant flooding as a function of injected PV.

After water flooding, 1 PV of the surfactant solution (0.025 wt % SDBS in brine) was injected as a reference case for tertiary recovery process. As shown in Fig 6, the use of surfactant shows negligible increment in oil recovery (2.1% of OOIP) after water flooding. After 0.5 PV of SDBS injections, oil production shows declining and plateau region was spotted on the curve after 0.7 PV and beyond, which indicates that no more oil could be displaced. Good or favorable mobility control is indicated by pressure gradients, as well as pressure drop trends can be used to confirm whether microemulsions were formed during the flood. As shown in Fig 6, the pressure drop indicates a continuous decrement which suggests the formation of no/weak microemulsion during the flood at high temperature. This agrees with a low viscosity of displacing fluid, having a viscosity ratio of 7.56 between crude oil and SDBS solution. Hence, the surfactant flooding leads to poor sweep efficiency which is a measure of the effectivies of an EOR process that depends on the volume of crude oil contacted by the injected fluid.

**Fig 7:**
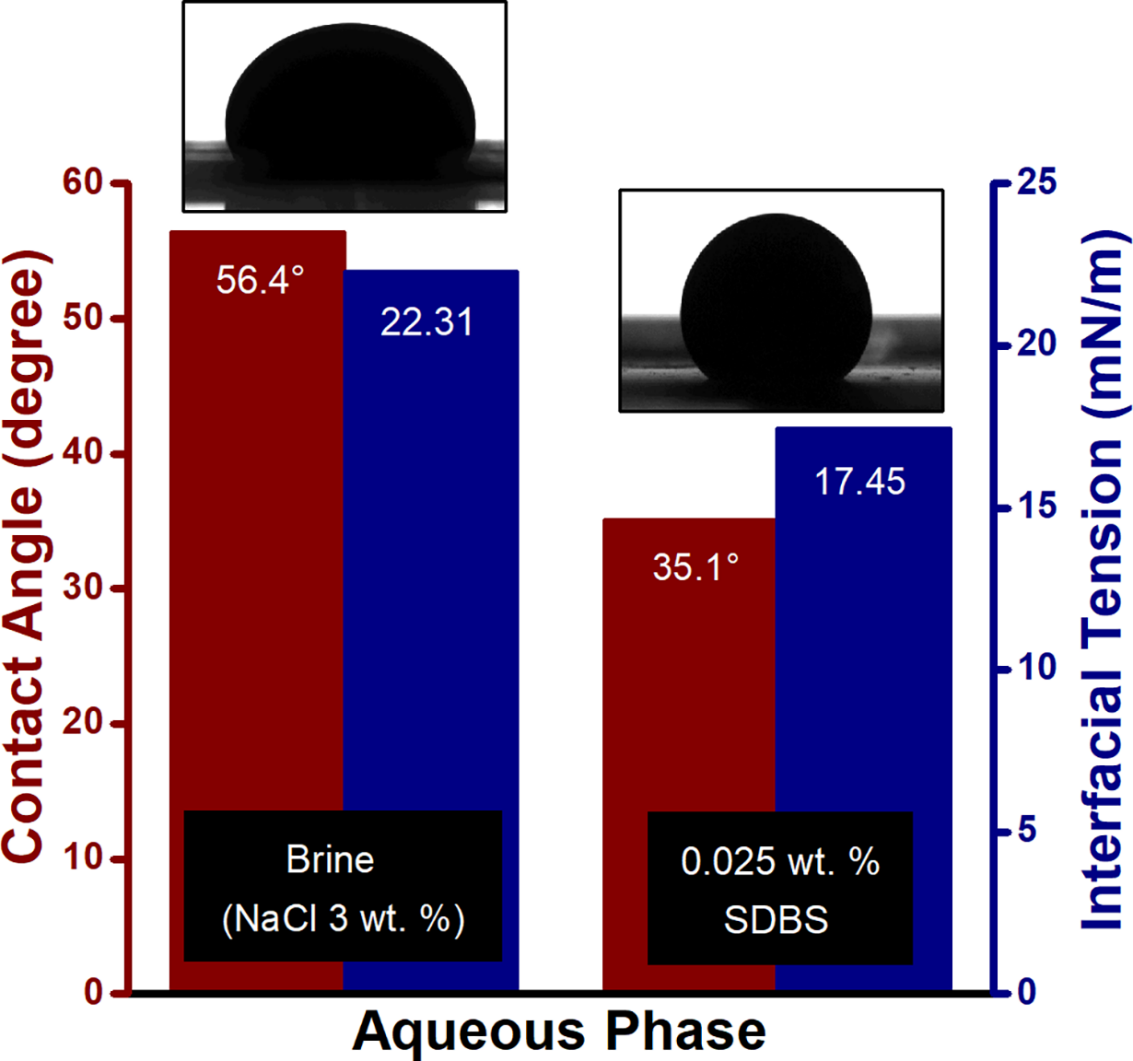
Interfacial tension and contact angle measurement for crude oil against brine and 0.025 wt. % SDBS at ambient condition.

The Fig 7 shows the reduction of interfacial tension between crude oil and surfactant solution, along with wettability alteration at room temperature. However, at high temperature (≥60°C), SDBS tend to degrade [34] which reduce the interaction between the surfactant and crude oil. Consequently, this SDBS degradation results in decrement of oil recovery during the surfactant flooding.

### Scheme II: Nano flooding

In this scheme, ZnO nanofluids were continuously injected for 1 PV as a tertiary recovery (Nano-EOR). For a particular temperature, the displacement efficiency (*E*_*D*_, %) due to Nano-EOR has also been evaluated, which depicts the fingering due to the loss of mobility control in the fluid flow [35]. The displacement efficiency is tabulated in Table 3, using formula as follows:

$$E_{D}=\left[1-(\frac{S_{or2}}{S_{or1}})\right]\times 100$$ (11)

where *S*_*or1*_ and *S*_*or2*_ represent residual oil saturation after brine injection (waterflooding) and Nano-EOR, respectively.

**Fig 8:**
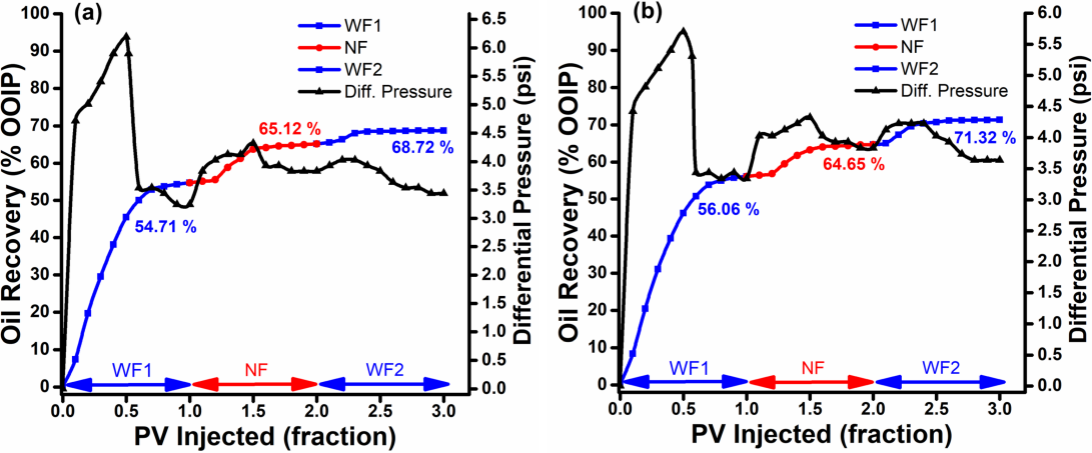
Cumulative oil recovery. Recovery performance vs. differential pressure of conventional nano flooding as a function of injected PV for (a) ZnO@500 NF and (b) ZnO@800 NF.

Both 0.1 wt. % ZnO NPs (500 & 800°C), dispersed in 0.025 wt. % SDBS under optimal stability conditions, successfully reduced residual oil saturation. They showed noticeably higher oil recovery than SDBS alone, with the additional recovery of 8.5–10.2% of OOIP as shown in Fig 8. However, the nanofluids needed a certain amount of time to displace additional oil during nanoflooding, possibly due to physicochemical interactions between the nanoparticles and the sandpack. The Fig 8 also showed that increasing nanoparticles size would decrease incremental oil recovery for ZnO@800 at relatively similar residual oil recovery. The highest additional oil recovery was achieved from smallest nanoparticle size. Similarly, Table 3 shows that the displacement efficiency due to Nano-EOR also increases as the nanoparticle size decrease.

**Fig 9:**
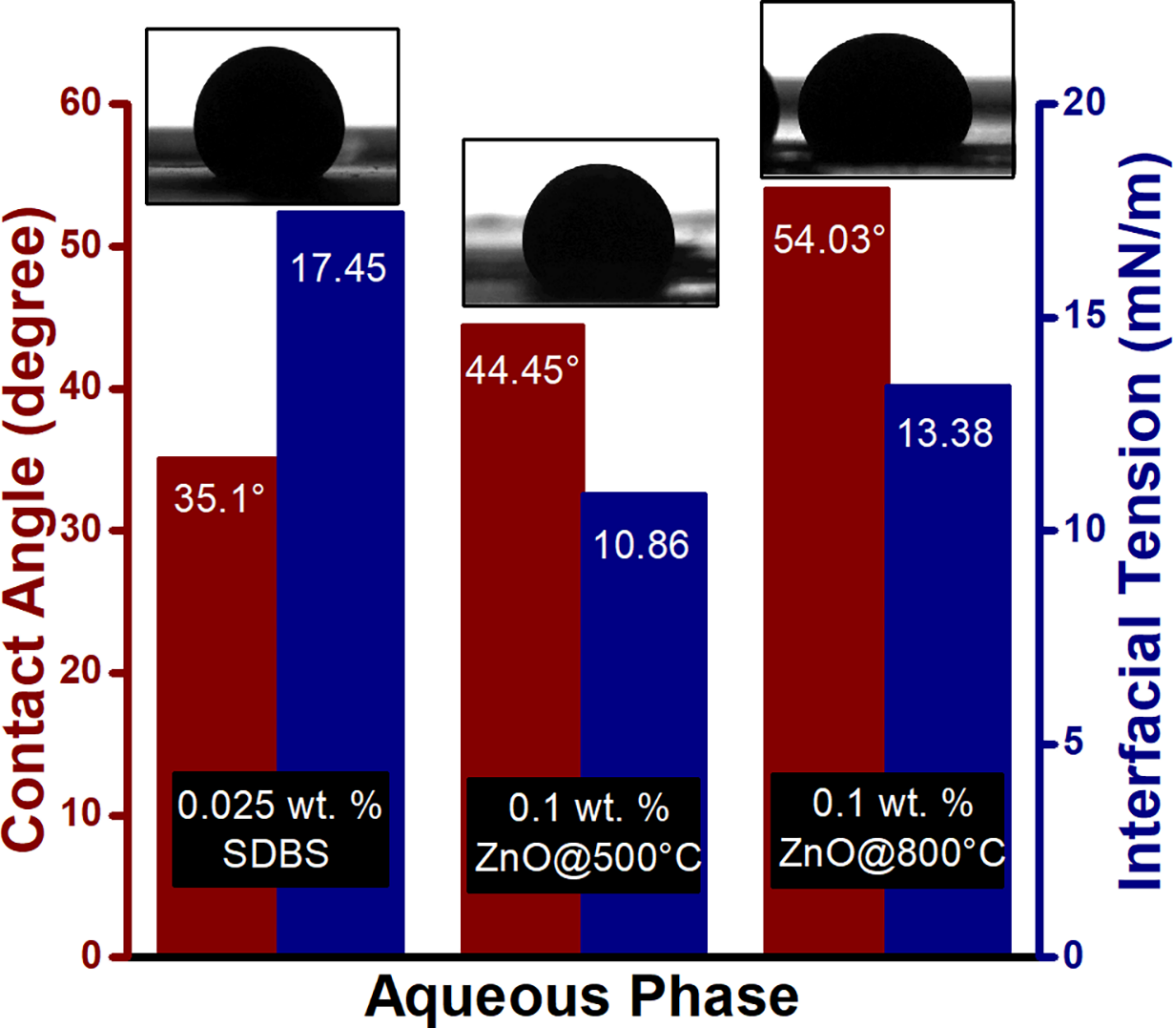
Measured interfacial tension and the contact angle of crude oil against ZnO NFs/SDBS at ambient condition, along with the captured images.

The increase in incremental oil recovery with the decrease in particle size is in accordance with the experimental results of IFT (as shown in Fig 9), which also decreased with the reduction in particle size of suspended ZnO NPs. The presence of NPs reduced the IFT between the oleic phase and the aqueous phase in the range of 13.38–10.86 mN/m, compare to 17.45 mN/m for SDBS alone. This reduction in IFT caused by the interaction of nanoparticles with the crude oil/SDBS interface due to a difference in tension (tension gradient). Meanwhile, the contact angle of ZnO NFs also deceased as the nanoparticle size decreases. However, the ZnO NPs in aqueous dispersion of SDBS increases the contact angle from 35.1° (for SDBS alone) to 54.03° and 44.45° for ZnO@800, and ZnO@500 NPs, respectively. This decrease in water-wetness can be explained by the fact that the ZnO NPs carries negative surface charges due to the SDBS adsorption, stay individually in the water phase and have the repellent force with the negative charge of the quartz plate [21]. Therefore, indicate a low adsorption capacity of ZnO NFs; as well as low retention in the porous medium. Morrow [36] observed that the oil recovery decreased with the decreasing water wetness, which is consistent with the notion that the strong wetting preference of the porous medium for water and associated high capillary forces provide the most efficient oil displacement. Hence, it can be established that the degree of IFT reduction plays a dominant role in oil displacement during ZnO nano flooding. Moreover, high temperature leads to the reduction of oil viscosity. This leads to the decrease in viscosity ratio (at 10 s^-1^) of oil and nanofluids from 18.65 (25°C) to 1.59 (95°C) and 16.44 (25°C) to 1.57 (95°C) for ZnO@500 and ZnO@800, respectively; since ZnO NFs are proven to be relatively stable at high temperature [24]. This ultimately reduced the mobility ratio which will divert injected NFs into unswept areas and consequently resulting in a favorable oil recovery. It is suspected that the incremental oil at high temperature was recovered not only due to the decreased mobility ratio, but also the decrement in IFT with increasing temperature since the molecular interactions between the liquids are weaker. Another possibility is the intensity of Brownian movement increases with the temperature of the medium and with the reduction of its viscosity and the particle size [37]. Since Brownian motions might be one of the energies that drive oil displacement due to nanoparticles, the force will increase. Overall, the combination of ZnO NPs and dispersant seems sigificantly promising in higher temperature, e.g., reservoir temperature.

In the last step (WF2), brine was re-injected to flush out any retained particles and have further decreased residual oil in the sandpack. As shown in Fig 8, the residual oil saturation of ZnO@500 NF decreased from 35 to 31.4% of PV, whereas the total oil recovery increased from 64.9 to 68.5% of OOIP. While in case of ZnO@800 (Fig 8B), the total oil recovered increases from 64.6 to 71.3% of OOIP. This suggests that the ZnO NPs potentially have the ability to displace more oil during WF2 stage, due to minimum particle trapping. Surface modified ZnO NPs carries negative surface charge, therefore, have a low adsorption capacity on negatively charged sand particles, and are easily washed-out. Hence, displace more oil. The nanoparticle adsorption through the porous medium involves physicochemical interactions between NPs and grain surfaces [38].

#### Pressure drop profile

The differential pressure was recorded by a pressure sensor (PS100, Lutron) with range 0–10 bar for the whole flooding period as shown in Fig 8. In the first PV of brine flooding, 2-phase flow is occurring so increase of differential pressure was observed. Once brine breakthrough, the differential pressure went down and stabilized at about 3.3–3.2 psi. After brine flooding, about 1 PV of nanofluid was injected into sandpack, while the differential pressure slightly increased. The reason may be that only few surface modified nanoparticles adsorbed and blocked pore channels due to low adsorption capacity; thereby somewhat reduced the permeability of sandpack. This also shows that the use of SDBS successfully minimizes the particle retention. Hence, the differential pressure steadily increased during the NF sequence. During the last sequence, the differential pressure decreased from the NF to the WF2 sequence. However, the pressure drop as low as the pressure drop during the WF1 sequence could not be attained.

### Scheme III: EM-assisted nano flooding

**Fig 10:**
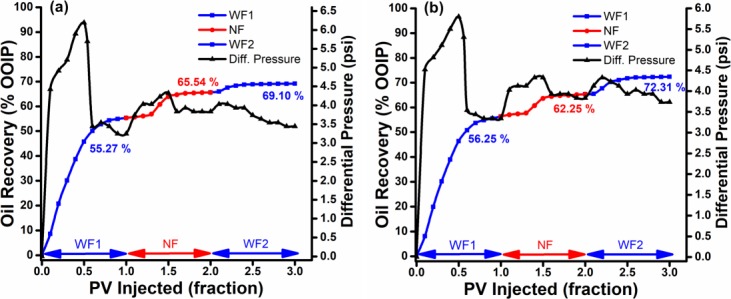
Oil recovery performance. Cumulative oil recovery and pressure drop profile as a function of injected PV for EM-assisted nano flooding of (a) ZnO@500 NF and (b) ZnO@800 NF.

The production performance of EM-assisted nano flooding is shown in Fig 10 and the results are summarized in Table 3. In case of EM-nano flooding, 10.4 and 9% OOIP additional oil recovered after water injection were observed for ZnO@500 and ZnO@800, respectively. Therefore, the additional recovery for EM-nano flooding is relatively higher than only nano flooding.

**Fig 11:**
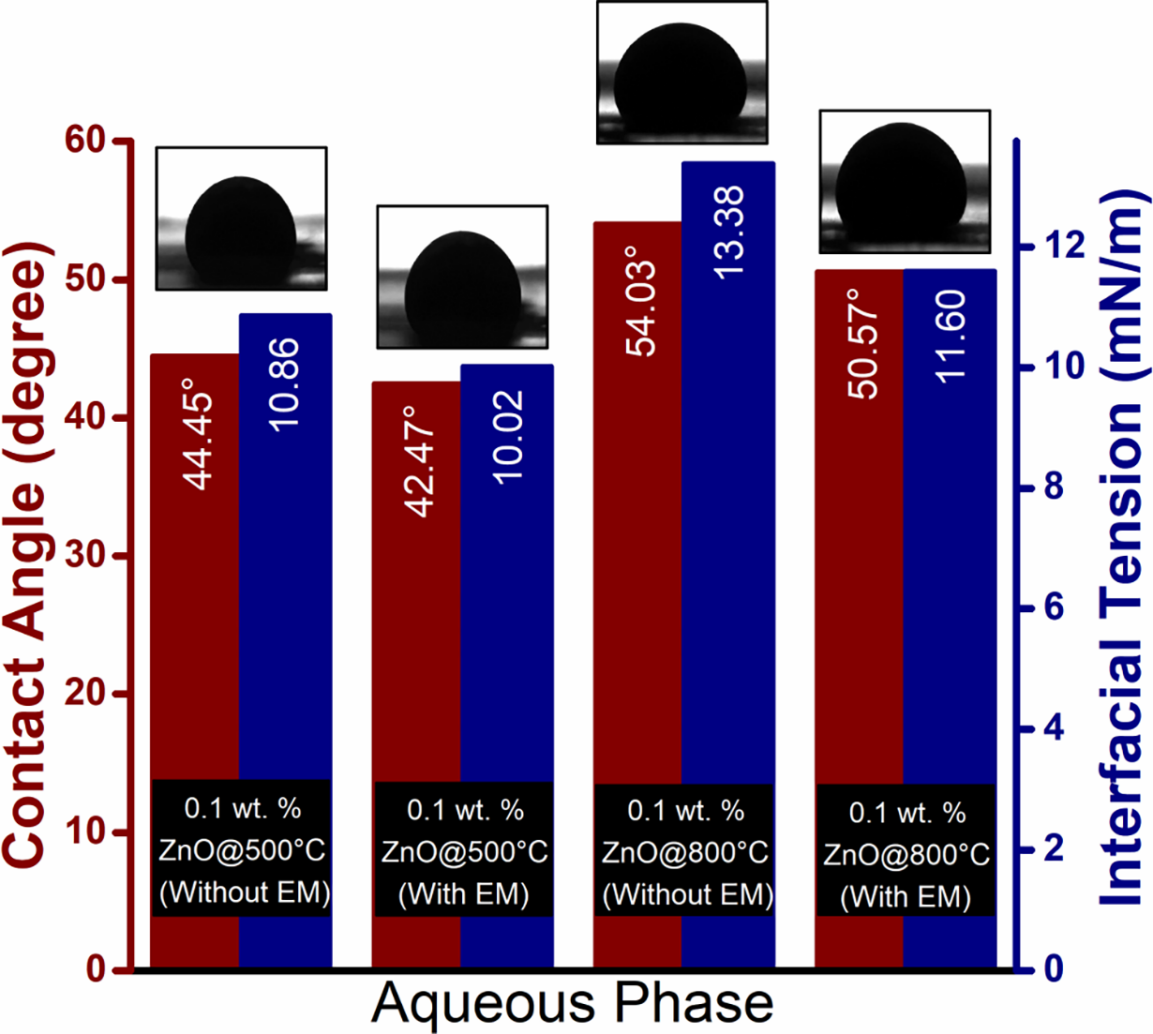
Effect of electromagnetic waves on interfacial tension and contact angle of crude oil with ZnO NFs/SDBS as the aqueous medium.

The increment in oil recovery under EM waves can be explained by different recovery mechanism as follows: (1) the dielectric polarization of nanoparticles causes the shape of oil drops to deform which increases the surface area for more particles’ adsorption, and consequently to a reduction in interfacial tension (Fig 11); (2) the rate of wettability alteration increases, leading to an increase in surface free energy (Fig 11); (3) the improvement of mobility ratio, due to electrorheological effect (ER) which increases the viscosity of ZnO nanofluids [21].

**Fig 12:**
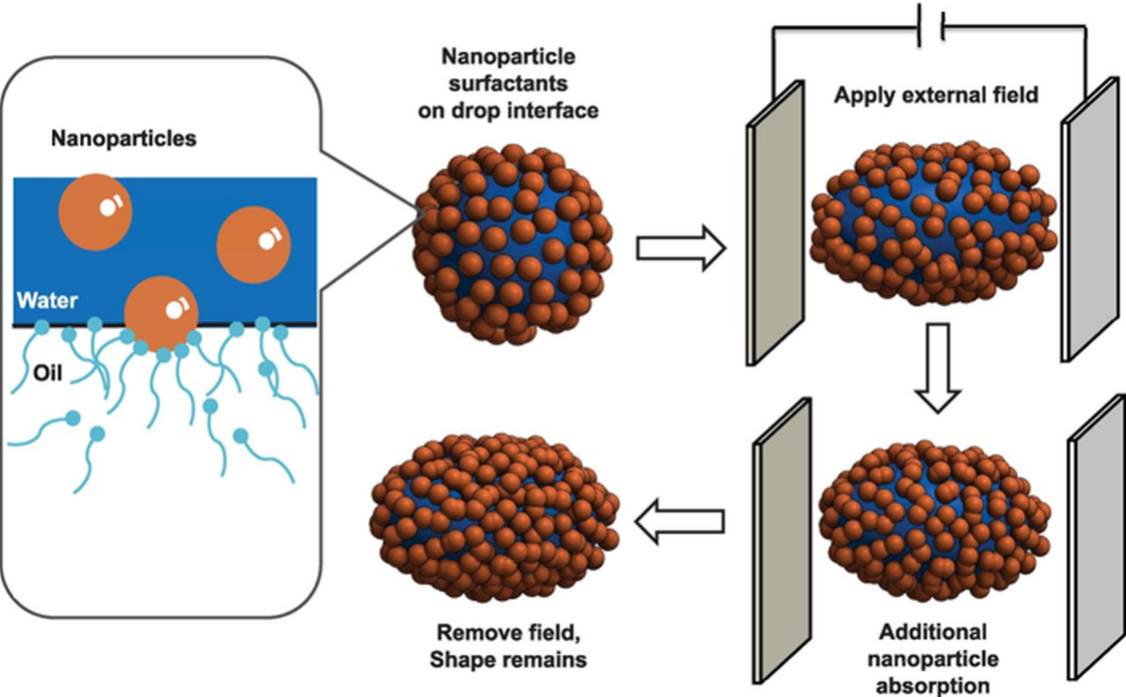
Schematic representation of deformation of oil drop, surrounded with nanoparticles, by an electric field.

For ZnO@500 NF (as shown in Fig 11), the IFT and contact angle value decreased from 10.86 to 10.02 mN/m and 44.45° to 42.47° respectively. While for ZnO@800 NF, the IFT value (contact angle) measured to be 11.6 mN/m (50.57°) under EM waves. Similarly, the viscosity ratio under EM waves show a decrement of 1.54 from 1.59 for ZnO@500; and 1.51 from 1.57 for ZnO@800 NFs. Therefore, it is reasonable to conclude that the rotational polarization of ZnO NPs at the interface causes the deformation of the oil droplet, which leads to the IFT reduction proportional to the additional oil recovery. The whole phenomenon of oil droplet deformation is illustrated in Fig 12.

The similar trend is observed, as in scheme 2, that increasing particle size (i.e. ZnO@800) will decrease incremental oil recovery. However, during the post-flush, ZnO@800 NF (Fig 10B) shows a greater incremental oil recovery of 7% OOIP, compared to 3.5% OOIP for ZnO@500 nanofluid. This indicates that the injected NFs have actually blocked the high permeable water channels due to ER effect, and reduce the mobility of the water phase. As a consequence, the subsequently injected brine (WF2) is diverted to the unswept area and leads to a high sweep efficiency and cumulative oil recovery.

#### Pressure drop profile

The pressure drop profile for EM-assisted nano flooding, as shown in Fig 10, depicted the similar pattern for brine flooding as in Fig 8. The pressure differential during the EM-nano flooding, however, shows a slight increase. This modest increase is ascribed to the formation of particles chain under EM waves, and the subsequent water channel blockage in the flooding process. The relationship of pressure drop and incremental oil recovery also proves that the increase in pressure differential is associated with an increase in oil recovery, which has been discussed in above section. At last sequent, the post-flush shows a slight increment in pressure drop as it enters the unswept zone. However, it soon started to gradually decrease once the re-injected brine flushes out the NPs from pore structure.

## Conclusion

A comprehensive laboratory study on zinc oxide nanoparticles for conventional, and EM-assisted nano-EOR were performed at a reservoir temperature of 95°C. Relationship between the IFT (crude oil/NF), contact angle (NF/quartz), viscosity ratio (crude oil/NF), and additional oil recovery were investigated to determine the possible oil recovery mechanism. It was observed that the oil displacement mechanism is dependent on the particle size of ZnO NFs, where the IFT and contact angle decreases with the decrease in particle size. These results are consistent and proportional to the additional oil recovery from the conventional nanoflooding experiment of ZnO@500 NF (55.7 nm), which shows the maximum oil recovery (10.27% OOIP) as well as displacement efficiency (22.96%). Meanwhile, ZnO@800 NPs (117.1 nm) show a greater change in oil recovery between conventional and EM-assisted nanoflooding. This can be the reason of larger disturbance in interface due to bigger particle size under orientational polarization, providing a noticeable reduction in IFT from 13.38 to 11.60 mN/m. Additionally, the low particle adsorption in the porous medium during transport process was identified from the pressure profile analysis; suggesting the minimum particle trapping inside the porous medium. This is in accordance with increased oil recovery during WF2 stage, especially in case of ZnO@800 NFs, i.e. 6.66% OOIP compare to 3.55% OOIP for ZnO@500 NFs. This also shows that the use of SDBS successfully minimizes the particle retention. The ER effect of ZnO@800 NPs also plays a significant role during WF2, which shows an increment in oil recovery from 6.66 to 7.05% by blocking the high permeable water channels. Hence allows the injected brine (WF2) to enter the unswept area, and consequently increase the oil recovery as well as the swept efficiency. Overall these results reveal a novel way to use water-based ZnO nanofluids for enhanced oil recovery purposed at a relatively high reservoir temperature.
